# Point-of-Care Urine Tests for Smoking Status and Isoniazid Treatment Monitoring in Adult Patients

**DOI:** 10.1371/journal.pone.0045913

**Published:** 2012-09-28

**Authors:** Ioana Nicolau, Lulu Tian, Dick Menzies, Gaston Ostiguy, Madhukar Pai

**Affiliations:** 1 Department of Epidemiology and Biostatistics, McGill University, Montreal, Canada; 2 Rollins School of Public Health, Emory University, Atlanta, Georgia, United States of America; 3 Respiratory Division, McGill University Health Centre & Montreal Chest Institute, Montreal, Canada; University of Cape Town, South Africa

## Abstract

**Background:**

Poor adherence to isoniazid (INH) preventive therapy (IPT) is an impediment to effective control of latent tuberculosis (TB) infection. TB patients who smoke are at higher risk of latent TB infection, active disease, and TB mortality, and may have lower adherence to their TB medications. The objective of our study was to validate IsoScreen and SmokeScreen (GFC Diagnostics, UK), two point-of-care tests for monitoring INH intake and determining smoking status. The tests could be used together in the same individual to help identify patients with a high-risk profile and provide a tailored treatment plan that includes medication management, adherence interventions, and smoking cessation programs.

**Methodology/Principal Findings:**

200 adult outpatients attending the TB and/or the smoking cessation clinic were recruited at the Montreal Chest Institute. Sensitivity and specificity were measured for each test against the corresponding composite reference standard. Test reliability was measured using kappa statistic for intra-rater and inter-rater agreement. Univariate and multivariate logistic regression models were used to explore possible covariates that might be related to false-positive and false-negative test results. IsoScreen had a sensitivity of 93.2% (95% confidence interval [CI] 80.3, 98.2) and specificity of 98.7% (94.8, 99.8). IsoScreen had intra-rater agreement (kappa) of 0.75 (0.48, 0.94) and inter-rater agreement of 0.61 (0.27, 0.90). SmokeScreen had a sensitivity of 69.2% (56.4, 79.8), specificity of 81.6% (73.0, 88.0), intra-rater agreement of 0.77 (0.56, 0.94), and inter-rater agreement of 0.66 (0.42, 0.88). False-positive SmokeScreen tests were strongly associated with INH treatment.

**Conclusions:**

IsoScreen had high validity and reliability, whereas SmokeScreen had modest validity and reliability. SmokeScreen tests did not perform well in a population receiving INH due to the association between INH treatment and false-positive SmokeScreen test results. Development of the next generation SmokeScreen assay should account for this potential interference.

## Introduction

In 2010 alone, there were an estimated 8.8 million incident tuberculosis (TB) cases and 1.5 million TB-related deaths globally [1]. Adherence to TB therapy is critically important to ensure cure and to reduce TB transmission. Adherence is major concern with treatment of latent TB infection (LTBI) which has a much more prolonged treatment period, typically nine months of isoniazid (INH), which often results in lower treatment completion rates. Currently, there is no perfect way of measuring adherence to TB medication, although a variety of approaches are used.

Smoking and TB have been found to have common epidemiological and clinical links [2]. There have been three recent systematic reviews that have shown that smoking is an independent risk factor for TB infection, TB disease, and TB mortality [3]–[5]. Globally, smoking rates have increased over the past three decades in developing countries [2]. The increase in smoking rates may potentially exacerbate the high TB burden in such countries [2]. Additionally, some studies suggest that adherence rates are lower in TB patients who smoke [2]–[7].

Rapid, point-of-care (POC) tests have been developed to monitor isoniazid (INH) TB treatment and smoking behaviours. These point-of-care tests, when used together, may help identify smoking patients who are at increased risk of being non-adherent to TB medication as well as at high risk of TB infection and disease. Once this high-risk group is identified, appropriate measures can be taken to help these patients complete treatment and quit smoking.

Two such relatively new point-of-care tests are IsoScreen® and SmokeScreen® (GFC Diagnostics, Oxfordshire, UK). IsoScreen is a newly developed point-of-care colorimetric test that detects INH metabolites in urine, and SmokeScreen detects cotinine metabolites in urine used to determine smoking status. The chemical assay developed for IsoScreen is based on the Arkansas colorimetric assay [6]. The Arkansas method produces a biochemical reaction that breaks the pyridine ring structure of INH and its metabolites, allowing the attachment of barbituric acid, and forming a coloured derivative that dyes the sample a blue-purple colour [6].

The SmokeScreen test is based on the König reaction [7]. The König reaction uses 1,3-diethyl-2-thiobarbituric acid as the condensing agent, and chloramine-T and potassium cyanide as the reactants [7]. The reacting agents break the pyridine ring structure of nicotine and its metabolites, allowing the attachment of the condensing agent 1,3-diethyl-2-thiobarbituric acid [7]. The attachment of the condensing agent results in a pink or orange solution, indicative of a positive result [7].

Although these tests are on the market, there are very few published data on their validity and reliability, and no studies have been conducted testing both tests in the same population [8]–[12]. Additionally, there is potential for cross-interference of the tests because of the similar pyridine ring structures of nicotine and INH. The possibility of cross-interference has not been adequately studied.

**Figure 1:**
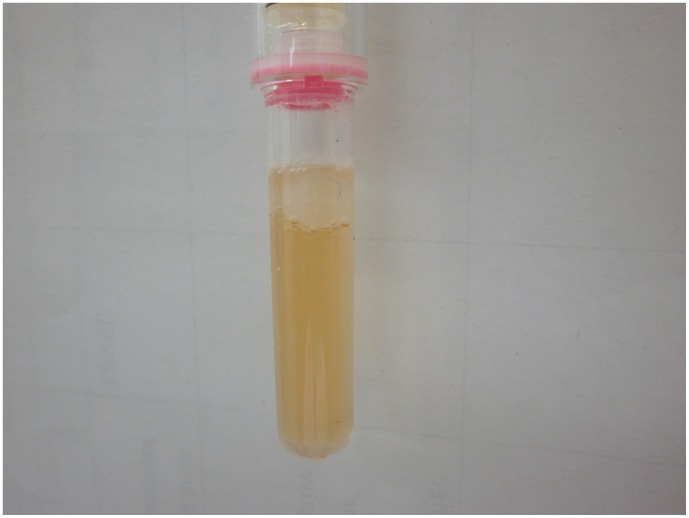
Example of a IsoScreen and SmokeScreen negative test result. Original urine colour (no colour change)  =  negative test result. Dark blue-purple IsoScreen test  =  positive test result. Orange-pink SmokeScreen test  =  positive test result. Any other colour change  =  indeterminate test result.

**Figure 2:**
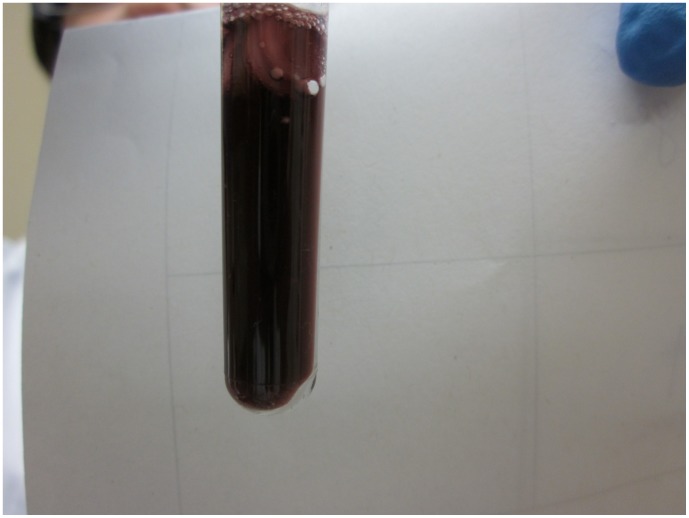
Example of a IsoScreenpositive test result. Original urine colour (no colour change)  =  negative test result. Dark blue-purple IsoScreen test  =  positive test result. Orange-pink SmokeScreen test  =  positive test result. Any other colour change  =  indeterminate test result.

**Figure 3:**
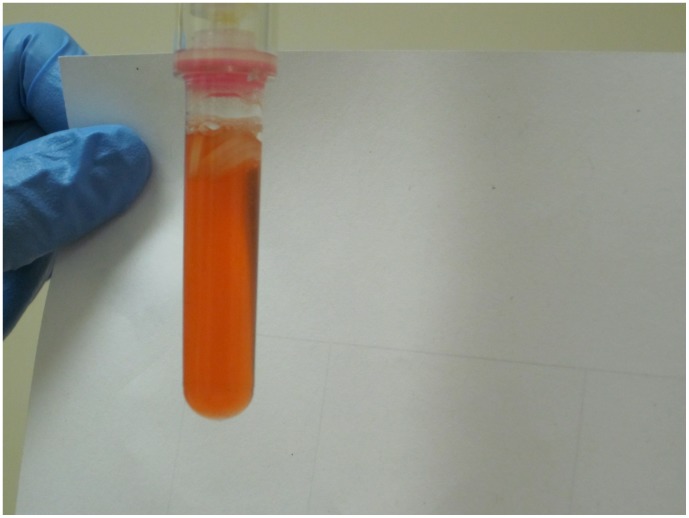
Example of a SmokeScreenpositivetest result. Original urine colour (no colour change)  =  negative test result. Dark blue-purple IsoScreen test  =  positive test result. Orange-pink SmokeScreen test  =  positive test result. Any other colour change  =  indeterminate test result.

## Materials and Methods

### Ethics Statement

All participants gave written informed consent, and the study was approved by the McGill University Health Centre (MUHC) Research Ethics Board, Montreal, Canada, in accordance to the principles expressed in the Declaration of Helsinki. Test kits were purchased from the manufacturer, and no funding support was sought from the company.

### Study Design and Setting

The study was a prospective comparison of IsoScreen and SmokeScreen tests to their respective reference standards. Outpatients from the tuberculosis clinic and/or the smoking cessation clinic were approached for enrolment from November 2010 to June 2011 at the Montreal Chest Institute (MCI), in Montreal, Canada. The MCI is a McGill university-affiliated teaching hospital that specializes in respiratory diseases and acts as a referral centre for active, latent, and probable TB cases, and contacts, in the province of Quebec.

In 2010, the active TB incidence in Montreal was estimated to be 7.5 cases per 100,000. At the MCI, the TB clinic cares for about 50 to 100 patients per week; these patients are investigated for TB, treated for latent or active TB, or are examined for immigration screening purposes. Data from a previous study [13] at the MCI have shown that a fifth of all outpatients attending the TB clinic were current smokers.

**Figure 4:**
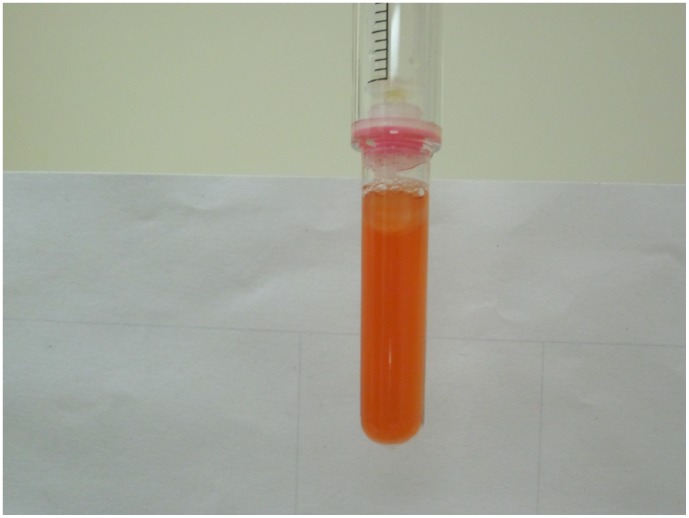
Example of an indeterminate IsoScreen result. Original urine colour (no colour change)  =  negative test result. Dark blue-purple IsoScreen test  =  positive test result. Orange-pink SmokeScreen test  =  positive test result. Orange IsoScreen test (not dark blue)  =  indeterminate test result.

**Figure 5:**
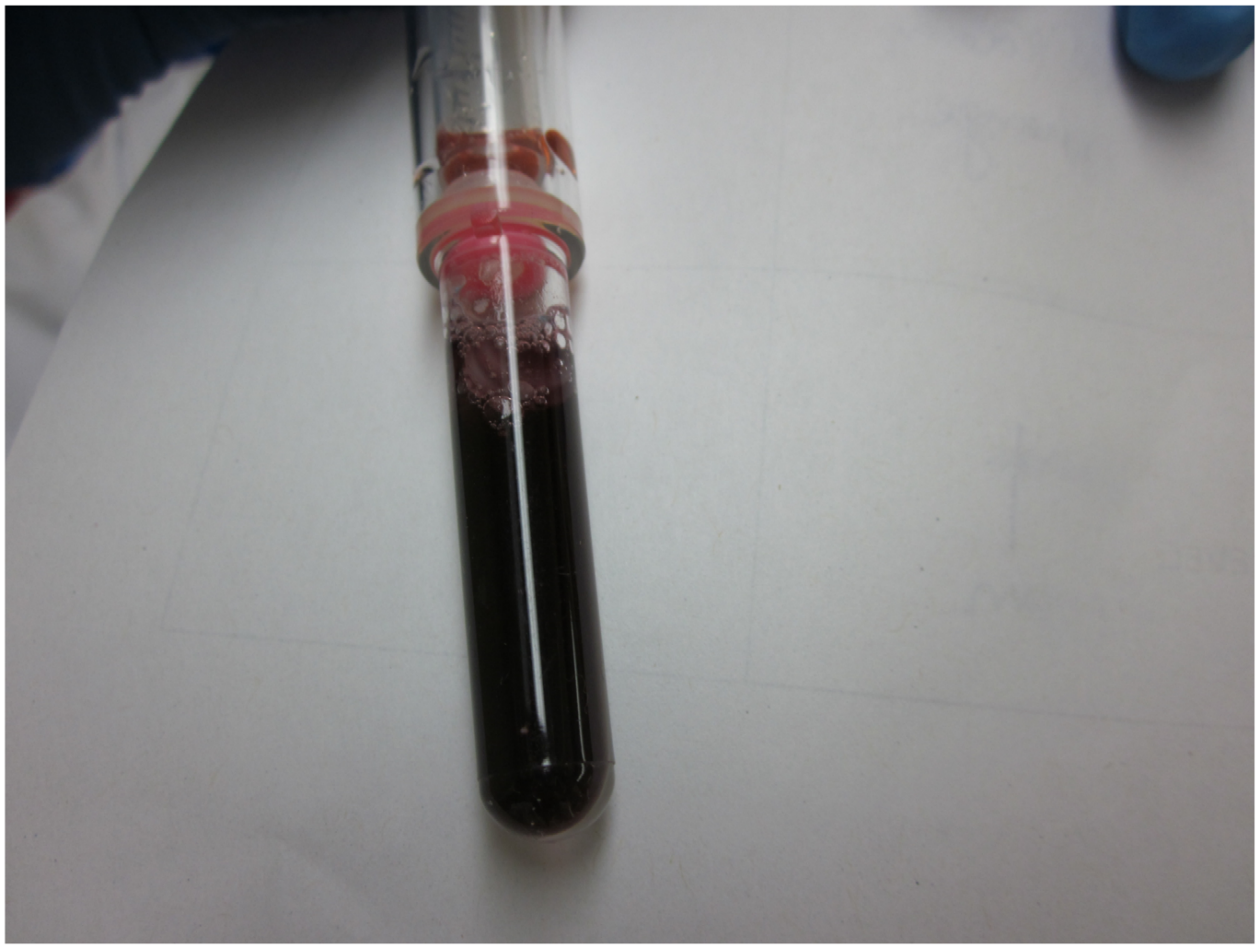
Example of an indeterminate SmokeScreen result. Original urine colour (no colour change)  =  negative test result. Dark blue-purple IsoScreen test  =  positive test result. Orange-pink SmokeScreen test  =  positive test result. Dark blue SmokeScreen test (not orange-pink)  =  indeterminate test result.

### Participants

Patients that met the following inclusion criteria were eligible to participate: at least 18 years of age, outpatients of the TB clinic and/or the smoking cessation clinic, and knowledge of the English or French languages. Patients were recruited from the outpatient TB clinic and the smoking cessation clinic, both located at the MCI. All patients were eligible for recruitment, regardless of whether they had active or latent TB or neither, or were on active TB or latent TB therapy. Patients were excluded if they were on nicotine replacement therapy, to prevent misclassification of smoking status.

### IsoScreen and SmokeScreen Tests

IsoScreen and SmokeScreen tests were performed according to the manufacturer’s instructions. These tests are made up of two mechanical parts: an upper syringe for drawing urine from the specimen, and a lower test tube-like reaction chamber. Testing was performed by mixing 2 mL of urine with the chemical tablet and waiting 5 minutes for the test to develop. A negative result by either test is shown by the original color of the urine sample (Figure 1). IsoScreen was considered positive if the test solution turned dark blue, purple, or green (Figure 2). SmokeScreen was considered positive if there was a colour change of pink, red, or orange (Figure 3). A colour wheel was provided by the manufacturer for interpreting SmokeScreen tests. The colour wheel was divided in five sections of various shades of orange and pink. The test colour of the urine was matched to one of the colours on the wheel. The colour wheel could also provide semi-quantitative information by matching different colour intensities (light to dark colours) to light, medium or heavy smoking. However, both tests were used in a dichotomous (positive/negative results) manner in the study.

In literature, an indeterminate result was defined as a non-positive, non-negative result [14]. In our study, indeterminate results were defined as any colour change that was not in the colour spectrum provided by the manufacturer’s package insert. For example, a colour change other than dark blue-purple for IsoScreen was deemed an indeterminate result. Similarly for SmokeScreen, a change in colour that did not correspond to orange-pink on the colour wheel was an indeterminate result (Figures 4 and 5).

According to the manufacturer’s product insert, IsoScreen can detect INH metabolites in urine for up to 30 hours after INH ingestion. The SmokeScreen test, on the other hand, can detect cotinine metabolites, which have a half-life of 20 hours and can be detected for days. All urine specimens collected were freshly voided and at room temperature before testing. The urine sample of every fifth participant was stored at room temperature to be used for reliability (reproducibility) testing.

### Reference Standards

Since there is no perfect method of identifying smokers and those on INH, we developed two reference standards, INH Intake Reference Standard Scale and Smoking Status Reference Standard Scale, based on composite information from multiple sources such as hospital charts and patient questionnaires. The INH Intake and Smoking Status Reference Standards were based on 5-point and 4-point scales.

Table 1 shows the scoring system we used for the INH Intake Reference Standard. This scale included treatment information from hospital records, directly observed INH intake, patient self-report, and urine INH levels measured with BD BBL Taxo® INH test strips (Becton, Dickinson and Company, New Jersey, USA). Directly performed INH intake was performed by the research nurse or research assistant with participants who had agreed to ingest their INH pill and had taken their last dose more than 18 hours prior to the clinic visit. The BD BBL test was performed as another measure of determining INH intake; the test strips were already being used as the routine test at the TB clinic for patients with active TB. BD BBL INH test strips were based on a modified assay by Kilburn et al. [15]. Studies suggested that INH test strips based on the Kilburn et al. assay had a sensitivity range of 93% to 99.5% and a specificity range of 96.4% to 100% [16]–[17]. To our knowledge, there have not been any studies validating the BD BBL INH test strips. Based on the information available, we made the assumption that the sensitivity and specificity of the BD BBL test was similar to that of the Kilburn assay.

**Table 1:** INH Intake Reference Standard scoring system.

Component	Criteria needed for points	Points allocated
Directly observed INH intake	Patient ingested INH tablet	+1
BD BBL Taxo test	Blue-purple or green positive results	+2
Hospital records	Prescription of daily INH	+1
Patient self-report	Questionnaire score was at least 40 points	+1
**Total points needed for positive INH intake classification**		**>2 pts**

Each of the four components was awarded a number of points based on how objective the measurement was. The BD BBL test was the most objective measure, thus it was awarded two points for test positivity. Directly observed INH intake was given one point because we expected few participants to have their INH pills with them and not to have taken their pill within 18 hours of the visit. The robustness of the scoring system was tested by changing the number of points allocated, but not the order of importance, and seeing if the validity and reliability measures changed.

Likewise, the Smoking Status Reference Standard Scale combined information on smoking status from hospital patient records, patient self-reported smoking status, and urine cotinine levels measured with NicAlert® (Nymox Pharmaceutical, Quebec, Canada). Studies that performed accuracy testing, comparing NicAlert to the gold standard gas chromatography for measuring current smoking versus non-smoking, found that the range of sensitivity measures was 97% to 99% and the range of specificity measures was 58.5% to 74.5% [18]–[22]. As shown in Table 2, we used a similar scoring system, with a participants being classified as a current smoker if the sum of the points was greater than 2.

**Table 2:** Smoking Status Reference Standard scoring system.

Component	Criteria needed for points	Points allocated
NicAlert test	Positive test- level 3 or higher	+2
Hospital records	Current smoker status recorded	+1
Patient self-report	Current smoker status	+1
**Total points needed for current smoker classification**		**>2 pts**

### Blinding

The research assistant performed the testing phase before filling out the case report form, such that the assistant was blinded to the patient INH and smoking information when reading the test results. The participant completed the questionnaire in a separate room while the testing was performed in a different room reserved for dealing with biohazardous material. All forms and samples were labelled with a study identification number. Two research assistants contributed to the data collection phase and both were trained at the same time using the study operating protocol. The data extraction form was clear and concise, with multiple choice or short answer questions to limit the variability between assistants in extracting data for the final reference standard components. The assistant performing the IsoScreen and SmokeScreen tests was not blinded while testing the NicAlert and BD BBL reference standard tests.

### Data Collection, Sample Size and Statistical Analysis

We collected patient demographic information, data on INH prescription practices, and smoking characteristics. Based on our planned recruitment of 200 participants and the expected SmokeScreen sensitivity of 89% and specificity of 98% [8], [10]–[11], the expected 95% CI for SmokeScreen sensitivity was between 83%–93% and between 94%–99% for specificity. Similarly, with an expected IsoScreen sensitivity of 95% and specificity of 98% [9], [12], [23]–[24], we estimated the 95% CI to be between 90%–97% forsensitivity and between 94%–99% for specificity.

Statistical analyses were performed using STATA 11 [25]. Sensitivity and specificity values were calculated using the method proposed by Simel et al. [14] that does not include indeterminate results in the calculations. Positive predictive values (PPV), negative predictive values (NPV), and positive and negative likelihood ratios (LR−, LR+) were also calculated.

Test reliability (reproducibility), measured by intra-rater and inter-rater agreement. The subsample used for reliability testing was comprised of every fifth participant that was in the study, to include a total of 38 individuals. Intra-rater variation was measured by doing two SmokeScreen tests and two IsoScreen tests on the same specimen, at the same time and with the same operator performing the tests. The purpose of testing the same specimen with two of the same tests was to test the amount of variability between the two test results, given a single reader.

Inter-rater variation was measured by having two separate testers that were trained using the same standardized training procedures, and performing the same test on the same specimen at the same time. Measuring inter-rater variation determined if the test produced the same results regardless of the person operating the test. Both operators were given standardized testing instructions.

Kappa statistic was calculated for inter-rater and intra-rater agreement. Intra-rater variation was calculated by testing two identical tests at the same time with the same operator and inter-rater agreement was between two tests at the same time with two operators.

Post-hoc, exploratory logistic regression models were done for SmokeScreen to explore possible covariates which could affect the number of false-positives and false-negatives. The covariates adjusted for in the multivariate regression model were: sex, age (continuous measure), foreign-born (yes/no measure), vitamin B6 intake (yes/no measure), INH treatment (yes/no measure), and the time interval from the last INH dose taken (continuous measure). This analysis was not performed for IsoScreen due to the low number of false-positive and false-negative results.

## Results

### Description of Study Participants

**Figure 6:**
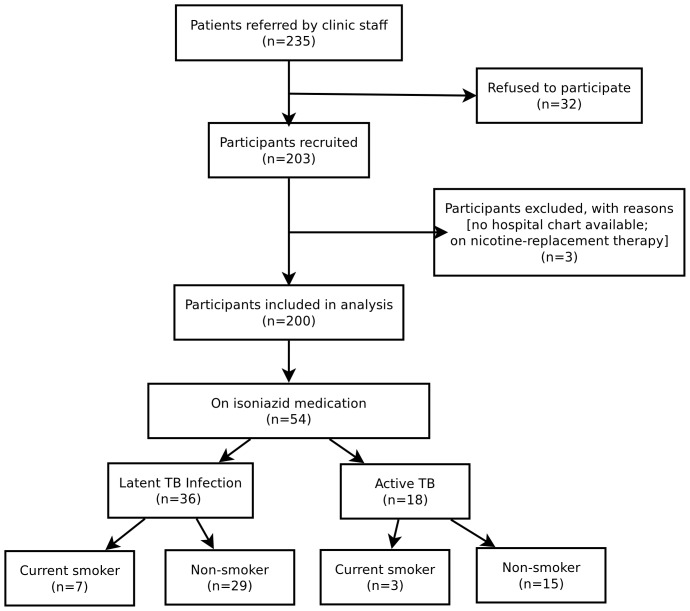
Flow chart of the study selection process, smoking status and isoniazid intake based on medical charts and questionnaire responses.

Of the 235 patients referred to the study by the attending physicians or the clinic staff, 200 (85.1%) participated in the study (Figure 6). As shown in Figure 6, 36 patients were treated with INH for LTBI and 18 patients were treated for active TB. Of the active TB patients, 3 were current smokers, and 7 were current smokers with LTBI. Participant characteristics are shown in Table 3. The median age was 40 years (range 18–80 years) and 51% of the participants (102) were male. About one third (58 [29%]) of participants were Canadian-born and 138 (69%) were from 52 different countries. Four (2%) participants were missing information on the country of birth. The second most common country of birth was Haiti, with 14 (7%) participants. 55 (27.5%) of participants were taking INH medication and about one third (65[32.5%]) were current smokers.

**Table 3:** Characteristics of participants obtained from medical records.

Characteristics	No. (% of N = 200) of participants
Men	102 (51.0)
**Age**	Mean 41.4 SD ±14.2; Median 40
18–20	7 (3.5)
21–30	43 (21.5)
31–40	53 (26.5)
41–50	39 (19.5)
>50	58 (29.0)
**Country of birth**	
Canada	58 (29.0)
Haiti	14 (7.0)
Algeria	13 (6.5)
Morocco	11 (5.5)
Other countries (49 different countries)	100 (50.0)
Not found	4 (2.0)
**Immigration Status**	
Citizens	113 (56.5)
Landed immigrants	71 (35.5)
Refugee claimants	14 (7.0)
Student visa	1 (0.5)
Not found	1 (0.5)
**Prescribed isoniazid medication**	
Yes	55 (27.5)
No	141 (70.5)
Not found	4 (2.0)
**Smoking status**	
Current smoker	65 (32.5)
Former smoker	21 (10.5)
Never smoker	108 (54.0)
Unknown smoking status	6 (3.0)

### Validity and Reliability of IsoScreen

We tested the three elements of the INH Intake Reference Standard Scale (BD BBL test, hospital records, and patient self-report) for between-component agreement. The Kappa statistic for the agreement between the three components (BD BBL test, hospital records, and patient self-report) of the INH Intake Reference Standard Scale was 0.7 [95% CI (0.6; 0.8)]. Directly observed INH intake was excluded from the between-component analysis because there was too much missing data.

In our study, the time interval from INH ingestion to urine collection varied from 30 minutes to 26 hours.

Summary measures of validity and reliability for IsoScreen are shown in Table 4. There were 4 (2%) indeterminate IsoScreen results, which were not included in the sensitivity and specificity analyses. The estimates of the sensitivity and specificity of IsoScreen were high at 93.2% [95% CI (80.3, 98.2)] and 98.7% [95% CI (94.8, 99.8)], respectively. Both PPV and NPV were good, with measures above 95%. The positive likelihood ratio (LR+) estimate was 70.8 [95% CI (17.8, 281.2)], and the negative likelihood ratio (LR−) estimate was 0.07 [95% CI (0.02, 0.2)]. The intra-rater and inter-rater variation κ statistics was fair to good, in the range of 0.6–0.75.

**Table 4:** Summary of validity and reliability measures for IsoScreen.

Analysis	Point estimate	95% Confidence Interval
Sensitivity (%)	93.2	80.3, 98.2
Specificity (%)	98.7	94.8, 99.8
Intra-rater variation, κ	0.75	0.48, 0.94
Inter-rater variation, κ	0.61	0.27, 0.90
Prevalence (%)	23.5	17.9, 30.1
Positive predictive value, PPV (%)	95.4	82.9, 99.2
Negative predictive value, NPV (%)	98.0	93.9, 99.5
Positive likelihood ratio, LR+	70.8	17.8, 281.2
Negative likelihood ratio, LR−	0.07	0.02, 0.2

### Validity and Reliability of SmokeScreen

The three components of the Smoking Status Reference Standard Scale (NicAlert test, hospital records, and patient self-report) were tested for between-component agreement. The Kappa statistic for the agreement between the three components was 0.8 [95% CI (0.8; 0.9)]. There were 10.5% (21/200) of SmokeScreen test results that were indeterminate.

Table 5 summarizes the validity and reliability of SmokeScreen. The sensitivity and specificity of SmokeScreen were low to moderate, with measures of 69.2% [95% CI (56.4, 79.8)] and 81.6% [95% CI (73.0, 88.0)], respectively. Similarly, the PPV and NPV were low to moderate. The LR+ value was 3.8 [95% CI (2.5, 5.7)], and the LR− estimate was 0.4 [95% CI (0.3, 0.5)].

**Table 5:** Summary of validity and reliability measures of SmokeScreen.

Analysis method	Point estimate	95% Confidence Interval
Sensitivity (%)	69.2	56.4, 79.8
Specificity (%)	81.6	73.0, 88.0
Intra-rater variation, κ	0.77	0.56, 0.94
Inter-rater variation, κ	0.66	0.42, 0.88
Prevalence (%)	34.5	28.0, 41.6
Positive predictive value (%)	68.2	55.4, 78.8
Negative predictive value (%)	82.3	73.7, 88.6
Positive likelihood ratio	3.8	2.5, 5.7
Negative likelihood ratio	0.4	0.3, 0.5

The Kappa statistic for intra-rater variation was good at 0.77[95% CI (0.56, 0.94)], and moderate for inter-rater variation at 0.66 [95% CI (0.42, 0.88)].

### Factors Associated with False-positive and False-negative SmokeScreen Results

Table 6 summarizes the results from the univariate and multivariate regression models, using the “false-positive tests” variable as the outcome for SmokeScreen testing. The following covariates were significant in the univariate models: age per 1-year increase [OR = 0.94 (95% CI: 0.91, 0.97]; foreign born with OR = 0.12 [95% CI (0.05, 0.29)]; vitamin B6 intake with OR = 12.2 [95% CI (3.5, 42.5)]; and INH treatment with OR = 97 [95% CI (30, 317)]. In the multivariate analysis, INH treatment remained significant with an OR of 57 [95% CI (16, 200)].

**Table 6:** Covariates associated with false-positive test results for SmokeScreen.

Covariate	Unadjusted model		Adjusted model†	
	OR	95% CI	OR	95% CI
Male	0.71	0.35, 1.45	0.60	0.18, 2.04
Age, years	0.94	0.91, 0.97	0.97	0.93, 1.02
Foreign born (yes/no)	8.38	3.47, 20.25	3.3	0.98, 10.84
Isoniazid treatment (yes/no)	97.42	29.91, 317.35	57	16.42, 200.52
Vitamin B6 intake (yes/no)	12.26	3.54, 42.47	1.6	0.31, 8.58
Time interval from last INH dose, hours	1.00	0.99, 1.01	1.00	0.97, 1.02

† Multivariate logistic regression model with sex, age, foreign born, B6 intake, INH treatment, and time interval from last INH dose as covariates.

Footnote: age and time interval from last INH dose are continuous variables; B6 intake, isoniazid treatment and foreign born are dichotomous yes/no variables.

Table 7 shows the results obtained from the univariate and multivariate regression models, using “false-negative tests” as a binary outcome. Age was the only significant covariate in the univariate model with OR = 1.04 [95% CI (1.01, 1.07)]. In the multivariate regression model age was no longer significant, but INH intake was significant with OR = 8.4 [95% CI (1.2, 61.2)].

**Table 7:** Covariates associated with false-negative test results for SmokeScreen.

Covariate	Unadjusted model		Adjusted model†	
	OR	95% CI	OR	95% CI
Male	0.48	0.19, 1.17	0.50	0.19, 1.27
Age, years	1.04	1.01, 1.07	1.04	1.00, 1.07
Foreign born (yes/no)	0.39	0.15, 1.03	0.44	0.14, 1.37
INH treatment (yes/no)	0.62	0.20, 1.91	8.43	1.16, 61.22
Vitamin B6 intake (yes/no)	0.59	0.07, 4.78	0.77	0.06, 9.91
Time interval from last INH dose, hours	0.83	0.65, 1.06	0.68	0.44, 1.05

† Multivariate logistic regression model with sex, age, foreign-born, B6 intake, INH treatment, and time interval from last INH dose as covariates.

Footnote: age and time interval from last INH dose are continuous variables; B6 intake, isoniazid treatment and foreign born are dichotomous yes/no variables.

## Discussion

IsoScreen and SmokeScreen have been previously validated in other studies; however, none of the studies tested the same population. There has only been one recently published study on IsoScreen in 2010 [9]. The remaining studies were published between 1996 and 2006, and they all validate either IsoScreen or SmokeScreen tests, but not both [8]–[12]. Two studies measured the IsoScreen sensitivity between 93% and 95% and the specificity at around 98% [9], [12]. Although these values are close to the manufacturer’s claims (97% and 98%, respectively), one of the two studies had the test developer as a co-author [12]. In addition, there were three studies that measured the sensitivity and specificity of SmokeScreen [8], [10]–[11]. The sensitivity had a wide range of 52% to 100%, which did not match the manufacturer’s claim (100%), but a specificity range (96% to 99%) closer to that stated by the manufacturer (98%).

We calculated validity and reliability measures by developing two composite reference standards for INH intake and smoking status. There was high agreement between the three out of four components of the INH Intake Reference Standard, and similarly for the agreement between all the components of the Smoking Status Reference Standard. This suggests that both reference standards had high reliability and we can be confident of our estimates of test accuracy.

Our study determined that IsoScreen had good validity and SmokeScreen had moderate validity. When comparing our results with values from the literature and with manufacturer’s claims, our sensitivity values corresponded to the lower end of the sensitivity range of other studies. However, compared to the manufacturer’s claims, the sensitivity we obtained was about 4% lower than their estimates. The specificity of IsoScreen was high, with the lower limit of the estimate being 94.8%. The estimates from other studies as well as the manufacturer claims were the same as the specificity we obtained.

Conversely, SmokeScreen had much lower sensitivity and specificity with values being 69.2% and 81.6%, respectively. The sensitivity we calculated was close to the lower end of the sensitivity range obtained from previous literature (52%). The SmokeScreen sensitivity we obtained was substantially lower than that of IsoScreen, and had wide confidence intervals. The SmokeScreen specificity was moderate, but 16% lower than the manufacturer’s claims and 14% lower than other published studies.

Based on the multivariate logistic regression, we found that treatment with INH was strongly associated with false-positive SmokeScreen results. We believe this association is due to the fact that INH and nicotine share molecular similarities. This was hypothesized before conducting the analysis and we thought the molecular similarities between nicotine and INH would interfere with the SmokeScreen chemicals in the test. However, there is no literature to show this interference exists. Taking B6 vitamins was also thought to produce false positive results but due to the imprecision of the estimate, B6 intake did not show significance. The multivariate analysis for false negative tests suggested that INH treatment was also positively associated with the number of false-negativeSmokeScreen test results.

Ideally, combined testing with IsoScreen and SmokeScreen tests should help clinicians focus treatment efforts on the smoking patients that are most at risk of non-adherence and have a high likelihood of poor treatment outcomes. However, IsoScreen on its own could not detect if the patient was taking the INH pill every day, for 6–9 months, as prescribed. IsoScreen can only be used at the point of care to determine if the patient has taken INH the day of the testing. It is difficult to infer from the IsoScreen results that the patient has been consistently following the entire course of treatment. Even though a negative test result can be helpful to determine if the patient is not taking the daily INH dose, a test that determines a more long-term adherence would be most helpful for health practitioners to manage patient treatment.

The results from this study suggest that IsoScreen and SmokeScreen tests should be used with caution when performed together in a TB clinic population because INH intake may cause false-positive SmokeScreen test results. Furthermore, other medications like rifampin can alter the normal urine colour, which may cause false-negative SmokeScreen results. Using the two tests in combination, at outpatient health centers similar to the clinics at the Montreal Chest Institute (MCI), might not be useful as tools to determine smoking TB patients at greater risk of non-adhering to medication.

However, IsoScreen was seen to have high sensitivity and specificity values and could be an effective tool to monitor INH intake. The test on its own may not be a good predictor of long-term TB treatment adherence. Therefore, we recommend using IsoScreen in combination with other commonly used methods such as prescription refill counts, pill counts, or self-reported adherence, in order to gain a better estimate of adherence.

Our study had several limitations. For both IsoScreen and SmokeScreen, inter-rater agreement was lower than intra-rater agreement. The low inter-rater agreement could be due to the operators not being blinded to each other’s results. We tested urine samples with four different tests: IsoScreen, SmokeScreen, BD BBL, and NicAlert. However, we only blinded operators from the results of the questionnaire but not from the results of all the tests. Evaluating IsoScreen and SmokeScreen with knowledge of the BD BLL and NicAlert test results would have artificially increased the sensitivity and specificity of the index tests. However, we did not rely only on the BD BBL and NicAlert results; we also used multiple sources of information to assess the reference standard. In addition, if such a bias was introduced we should expect both IsoScreen and SmokeScreen tests to perform similarly well. This was not the case.

Other limitations pertain to the composite reference standards. Although the agreement between the components of our scale was high, we do not know whether adding more pieces of information increases the overall validity of the tool. There was more chance of bias with every item added to the reference standard composite. For example, the medical charts used to collect INH intake and smoking status information were at times incomplete and outdated.

Simultaneous testing by the two operators was not always performed right after the urine sample was obtained. Moreover, reading the coloured results of the tests was a subjective method, as one colour could be easily construed as another shade, and the tests were judged according to a colorimetric scale. For example, this was the case with the SmokeScreen colour wheel (positive test indicated bycolour change of pink, red, or orange), which was at times very similar to the colour of the urine. If participants were also taking rifampin, which makes the urine an orange colour, it was very difficult to determine a change in colour for SmokeScreen.

The low sensitivity of SmokeScreen could be explained by the fact that it was hard to distinguish between the colour of the urine and the yellow-orange positive colour spectrum of the test. Since the patients in our study were also taking other medications at the same time as our testing, this could have led to misclassification of the smoking status. The test could also have difficulty detecting light traces of nicotine and may not be a good measure of occasional smoking, although this was not tested in our study.

In conclusion, the results of this study, although not generalizable to all settings, provide valuable information on the possible cross-interference of INH and smoking status testing and how this may affect the interpretation of point-of-care urine tests. This should inform the development of improved POC tests for TB treatment monitoring and smoking status. Future studies should also go beyond sensitivity and specificity, and assess whether using these rapid POC tests can actually improve clinical care by changing the decisions of doctors and patients.
